# Exploring needs, preferences and cultural influences on HIV/AIDS self-management among women of reproductive age in Northern Ghana

**DOI:** 10.4102/hsag.v31i0.3362

**Published:** 2026-10-07

**Authors:** Alhassan B. Adam, Nombulelo Sepeng, Fhumulani M. Mulaudzi, Maurine R. Musie

**Affiliations:** 1 Department of Nursing, School of Nursing and Midwifery, University for Development Studies, Northern Region, Tamale, Ghana; 2 Department of Nursing Science, Faculty of Health Sciences, University of Pretoria, Pretoria, South Africa

**Keywords:** HIV/AIDS, self-management, women of reproductive age, stigma, PEN-3 cultural model, Ghana

## Abstract

**Background:**

Women in sub-Saharan Africa experience a disproportionate burden of HIV/AIDS. Self-management of HIV is influenced by sociocultural and economic factors that shape adherence and well-being.

**Aim:**

To explore and describe the perception of women regarding their needs, preferences and cultural practices influencing self-management of HIV/AIDS in Northern Ghana.

**Setting:**

Antiretroviral therapy (ART) clinics in three main hospitals in Northern Ghana: Tamale Teaching Hospital, Northern Regional Hospital and Tamale West Hospital.

**Methods:**

A qualitative, exploratory and descriptive design was utilised for this study. Twenty women were purposively recruited. Data were collected using semi-structured in-depth interviews. Thematic analysis, guided by Braun and Clarke’s six-step framework was conducted with NVivo 15.2 to organise codes and themes.

**Results:**

Analysis within the perception, enablers, and nurturers (PEN-3) model identified: (1) perceptions of needs for self-management: knowledge of HIV disease process, consistent access to ART, well-organised clinic services and preference for long-acting injectables to reduce pill fatigue; (2) enablers: credible communication channels (mosques, churches, community durbars, festivals, local language media) and moderated digital supports such as WhatsApp reminders and clinic micro-lessons; (3) nurturers: comprised partners, family members, trained peers and digital peer networks that provided reminders and encouragement.

**Conclusion:**

Self-management of HIV/AIDS among women in Northern Ghana extends beyond medication adherence to include psychosocial and cultural dimensions.

**Contribution:**

Identifying women’s needs and preferences for HIV/AIDS self-management, and how religion, sociocultural and contextual factors uniquely shape their ability to manage their condition, providing evidence for culturally relevant, women-centred education programmes.

## Introduction

Self-management of HIV/AIDS extends beyond consuming antiretroviral medication, but is deeply influenced by social expectation, cultural norms and practices, and religious beliefs (Areri, Marshall & Harvey 2020; Dunn Navarra et al. 2020). Self-management refers to the ability of individuals to manage the symptoms, treatment, physical and psychosocial consequences, and lifestyle changes inherent in living with a chronic condition such as HIV/AIDS (Khumsaen & Stephenson 2019). Among women of reproductive age (WRA) living with HIV/AIDS, effective self-management practices include adherence to antiretroviral therapy (ART), positive lifestyle modification, disclosure, stigma management and access to culturally relevant psychosocial support (Areri 2021; Poe et al. 2020). In Northern Ghana, where traditional healing practices, communal living and religious observance are deeply rooted, culture and religion play a crucial role in shaping the health-seeking behaviours (Fatima, Naledi & Mbalenhle 2025) of WRA, including the decision concerning HIV/AIDS self-management, disclosure and treatment adherence. Despite the numerous studies that have explored adherence to ART in Ghana, little is known about the needs, preferences, and how cultural practices and religious beliefs shape women’s daily self-management practices. Women of reproductive age living with HIV in Ghana face multifaceted challenges in managing their HIV conditions, influenced by their needs, preferences and the interplay of socioeconomic, cultural and religious factors (Adongo et al. 2025; Boakye & Adjorlolo 2023). Studies have shown that limited knowledge, poverty, stigma, discrimination and limited access to healthcare services restrict WRA living with HIV/AIDS in Northern Ghana from adhering to ART treatment, and adopting effective self-management practices (Aivadyan et al. 2025; Dzansi, Tornu & Chipps 2020). Effective self-management includes: women’s empowerment, autonomy, disclosure skills, self-advocacy, participating in community-based support groups, medication adherence linked to daily religious routines, family and kinship engagement in HIV care, integrating traditional and biomedical care with HIV guidelines, and dietary and lifestyle adjustment respectful of local customs. Women of reproductive age prefer strategies and interventions that emphasise empowerment, autonomy and cultural appropriateness. Ashaba et al. (2024) found that disclosure skills, empowerment and self-advocacy were critical in shaping HIV self-management among young women in Uganda. The World Health Organization (WHO 2022) guidelines recommend self-care and self-management strategies to enhance reproductive health decision-making among WRA. Research consistently highlights the centrality of education and tailored self-management education programmes (SMEPs) in equipping women to manage HIV effectively (Obeagu & Obeagu 2024; Rebchook et al. 2022). Furthermore, cultural adaptation and community engagement have been identified as enablers of effective self-management practices (Valdiserri & Holtgrave 2020). In Botswana, the ‘Mothers Moving Towards Empowerment’ intervention utilised cognitive behavioural approaches to reduce stigma and improve social support (Poku et al. 2020). In the United States, nurse-led culturally tailored programmes for African American mothers have improved retention in care and health outcomes (Cheptoek et al. 2024). Within Ghana, religious and cultural norms remain central to health-seeking behaviour, and integrating these into HIV education could strengthen adherence and reduce stigma (Bergman et al. 2023; Kwena et al. 2023). The HIV epidemiological pattern in Northern Ghana highlights the need to explore beyond biomedical treatment approaches and consider the sociocultural dimensions that influence women’s self-management practices. Cultural practices and religious beliefs can be enablers (support) of self-management practices among women (Fauk et al. 2021). The interplay of culture and religion in Northern Ghana underscores the need for developing culturally appropriate, stigma reduction and gender-sensitive strategies that respond to women’s expressed needs and preferences while addressing structural barriers. Even with the growing number of women living with HIV/AIDS in low-income countries, very little research on culturally appropriate SMEPs for WRA is conducted in this setting (Dadi et al. 2024), Ghana in particular, where sociocultural and religious factors are deeply pronounced (Abdulai et al. 2022). This article emanates from an umbrella study towards the development of culturally appropriate SMEPs for WRA living with HIV/AIDS in Northern Ghana. There is a national and global recognition that self-management is pivotal for HIV/AIDS management (Dadi et al. 2024). In Ghana, recent estimates indicate that about 220 000–230 000 WRA are living with HIV (Appaw 2018). Globally, women account for more than half of all people living with HIV, and WRA account for a substantial proportion of new infections (WHO 2022). Sub-Saharan Africa continues to bear the highest burden, accounting for nearly two-thirds of global infections, with young WRA at increased risk because of biological, structural and sociocultural vulnerabilities (Boakye & Adjorlolo 2023; Mwendwa 2024; UNAIDS 2022). Studies highlight that sociocultural and religious contexts profoundly shape HIV/AIDS self-management. In Kenya, for example, Copeland (2018a) showed how cultural competence and consonance facilitated improved HIV/AIDS care and medication adherence among women. Similarly, in KwaZulu-Natal, women employed traditional remedies and family support systems as integral elements of self-management (Gumede et al. 2022). In Iran, structural stigma and economic insecurity constrained women’s ability to adhere to therapy (Bazrafshani et al. 2022). In North America, African American women reported intersecting stigmas and distrust in healthcare systems, further complicating adherence (Hassan & Coon 2024; Zuniga et al. 2018). These cross-regional findings underscore the need for interventions that are both culturally appropriate and context-sensitive. Understanding women’s perception about their needs and preferences within these strategies and interventions, particularly in culturally varied settings such as Northern Ghana, is critical. As a result of these findings, the study is underpinned by PEN-3 cultural model (Airhihenbuwa, Ford & Iwelunmor 2014), utilising the first domain (relationship and expectation), representing perception, enablers and nurturers. The interplay of these three sub-domain types can facilitate or hinder self-management behaviours among WRA living with HIV/AIDS. Despite these advances, most HIV self-management programmes prioritise biomedical and behavioural elements, with limited focus on cultural and religious practices that are deeply embedded in women’s lived experiences and perceptions in Northern Ghana. This region, characterised by high HIV prevalence and entrenched sociocultural traditions, provides a unique setting to explore how women perceive their needs and preferences regarding self-management (Copeland 2018b; Mwendwa 2024). Understanding these perceptions is crucial to designing interventions that are contextually appropriate, gender-sensitive and sustainable. Critically, identifying and addressing these disruptions is the most fundamental reason for conducting an in-depth exploration of women’s needs, preferences and cultural practices in relation to self-management. Therefore, this study aimed to explore and describe the perceptions of WRA living with HIV/AIDS in Northern Ghana regarding their needs, preferences and cultural factors influencing their self-management abilities. The development of SMEPs tailored to the needs, preferences and culture of women has been recommended by the WHO (2022). Findings from this study are expected to inform the design of culturally appropriate SMEPs that are tailored towards addressing women’s holistic needs and improving HIV care outcomes in similar contexts.

## Theoretical model

The PEN-3 cultural model (Airhihenbuwa 1995; Airhihenbuwa et al. 2014) (Figure 1) guided data collection and analysis, ensuring that cultural values, beliefs and practices were central to the interpretation of findings. The conceptual model for this study, as shown in Figure 1, focuses on three interwoven domains: *relationship and expectations, cultural empowerment and cultural identity*. The conceptual model is relevant in exploring, describing and understanding health-related issues within the specific cultural context of Northern Ghana. The paper focuses on the first domain of PEN-3 model: relationship and expectation. In relationships and expectations, the PEN represents individuals’ *perceptions*, which can either facilitate or impede health-related behaviours and choices. Additionally, it encompasses the *enablers* that have the potential to either encourage or discourage the adoption of healthy behaviours and practices. Lastly, it includes the *nurturers* who offer support by reinforcing or discouraging certain behaviours.

**FIGURE 1:**
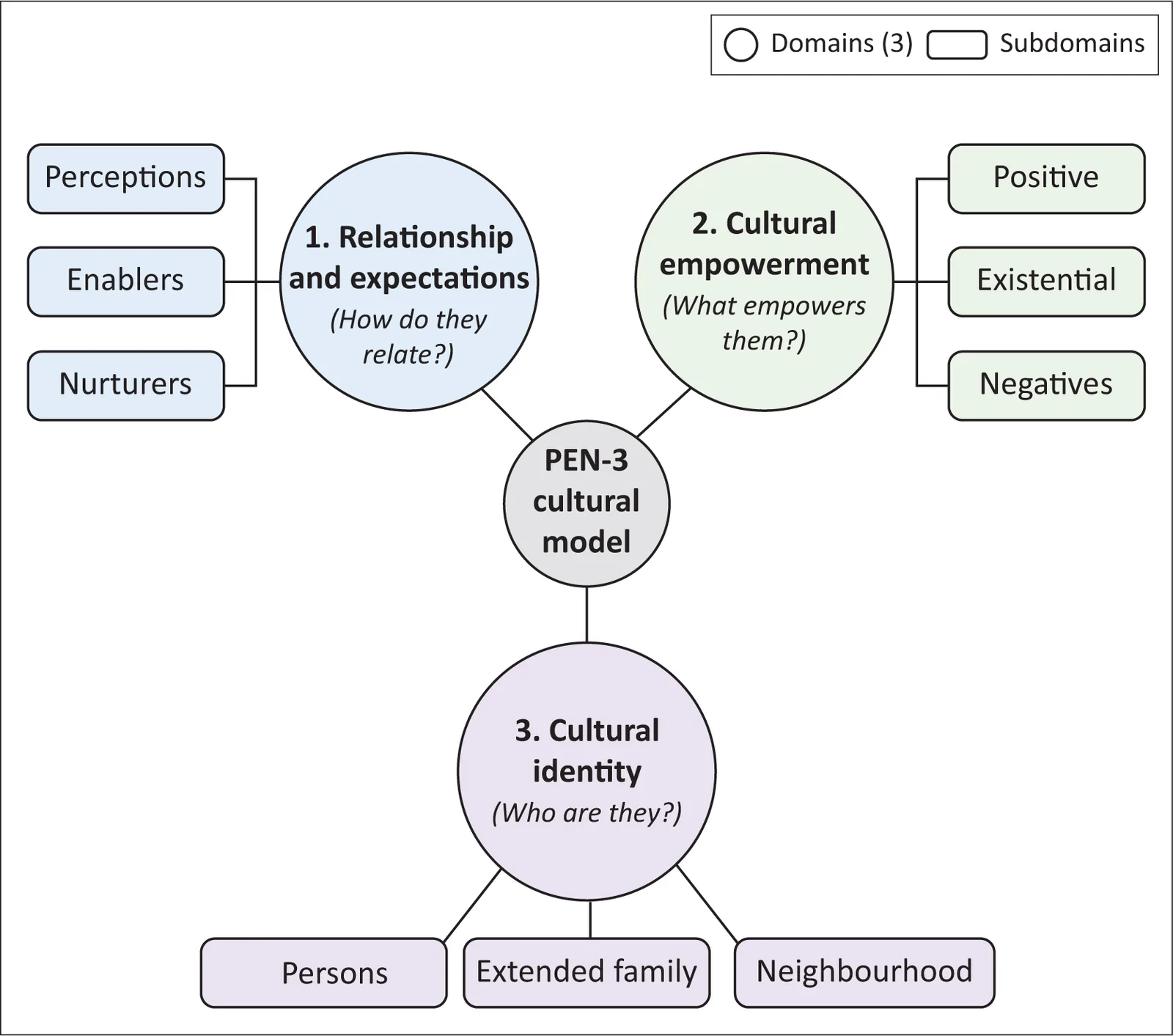
Illustration of the PEN-3 cultural model domains and subdomains, including relationship and expectations, cultural empowerment and cultural identity.

## Research methods and design

### Research setting

The study was conducted at three major healthcare facilities in the Northern Region of Ghana: Tamale Teaching Hospital (TTH), Northern Regional Hospital (NRH) and Tamale West Hospital (WH). These facilities provide comprehensive HIV/AIDS care, including counselling, testing and ART services, and serve as referral centres for surrounding districts. The Northern Region is predominantly rural, with strong traditional and religious structures that influence healthcare-seeking behaviours, making it a relevant area for exploring culturally appropriate HIV/AIDS self-management strategies.

### Design

The study employed a qualitative exploratory design to investigate the perceptions of WRA living with HIV/AIDS in Northern Ghana regarding their needs, preferences and cultural practices influencing HIV self-management. A qualitative exploratory design was considered appropriate because it facilitates an in-depth understanding of participants’ lived experiences, perceptions and meanings within specific sociocultural contexts (Khawaja, Muhammad & Siddiqui 2022).

### Study population and sampling strategy

The study population comprised WRA (15–49 years) who had a confirmed HIV/AIDS diagnosis and were routinely attending HIV/AIDS clinics at one of the three selected hospitals. Inclusion criteria were: (1) being female, aged 15–49 years, (2) confirmed HIV-positive status and (3) active attendance at ART clinics for counselling and treatment. Exclusion criteria were: (1) those women who are sick on the day of the interview or mentally unstable and (2) those who were irregular or non-attendees of HIV/AIDS clinics at the study sites. A total of 20 WRA (15–49 years) participated across the three study sites (TTH, NRH and WH). The participants varied in marital status, parity and duration of HIV/AIDS diagnosis. Data saturation was reached after the 18th interview, with two additional interviews conducted to confirm thematic adequacy. Purposive sampling was employed to recruit participants who could provide rich, relevant and diverse insights into the research objectives (Campbell et al. 2020). Healthcare providers, including nurses and physicians in ART clinics, supported recruitment by identifying eligible women. A sample size of 20 participants was selected, consistent with qualitative research standards that emphasise data saturation rather than statistical generalisability (Ahmed 2025). Data collection was discontinued once data saturation was achieved and no new themes emerged.

### Data collection

Data collection took place between 15 June and 15 July 2024. After obtaining institutional approval, hospital authorities at the three facilities facilitated entry into the HIV clinics. Eligible participants were approached through ART clinic nurses, informed of the study purpose and invited to participate. Written informed consent was obtained prior to the interviews. A pilot study involving three participants (one from each facility) was conducted prior to the main data collection to refine the interview guide and procedures. Feedback from participants and reflections by the research team were used to clarify ambiguous questions, improve the flow of interviews and enhance cultural sensitivity. Pilot data were excluded from the final analysis. In-depth, semi-structured interviews were conducted face-to-face in private settings within the clinics to ensure confidentiality. Interviews were conducted in English or Dagbani (local languages), depending on participant preference, with interpreters engaged when necessary. Each interview lasted approximately 45 min – 60 min and was audio-recorded with participants’ consent. Additionally, the lead researcher maintained detailed field notes using pen and notepad, documenting non-verbal cues, contextual observation and immediate reflections throughout the interview.

### Data analysis

Audio recordings were transcribed verbatim and cross-checked for accuracy against the recordings of the interviews conducted in English language based on the participants’ preference. Although some participants did not have formal education, they could speak English fluently. Data were analysed thematically following the six phases outlined by Braun and Clarke (2006): familiarisation with data, generating initial codes, searching for themes, reviewing themes, defining and naming themes, and producing the report. Coding was both inductive (emerging directly from the data) and deductive (guided by the PEN-3 cultural model). NVivo software (version 15.2) was used to facilitate coding, organisation and retrieval of data. Trustworthiness was ensured through triangulation of data sources (different hospitals), member checking, peer debriefing and maintaining an audit trail. The researchers ensured reflexivity and maintained an audit trail throughout the study by keeping detailed field notes and reflective notes to document the researchers’ decisions, assumptions and interactions during data collection and analysis. These strategies enhanced the transparency and trustworthiness of the study, with the lead researcher explicitly documenting positionality and potential biases.

### Trustworthiness

*Trustworthiness* of the study was ensured via the application of established qualitative rigour criteria, which include credibility, dependability, transferability and confirmability. *Credibility* was ensured through the use of a trained female nurse with postgraduate training in qualitative research as the primary interviewer, supported by two trained research assistants with nursing and public health backgrounds. Their professional experience and training enhanced effective probing and data collection. *Dependability* was ensured by adopting a consistent interview approach guided by the interview guide throughout the data collection process. Proper documentation of the research process and detailed field notes also enhanced consistency and reliability. *Confirmability* was achieved through maintenance of field notes, reflective observation and secure storage of audio recordings and transcripts, which granted the findings to be grounded in participants’ viewpoints rather than researcher bias. Also, the authors further ensured confirmability by ensuring that interviews were conducted in private locations that were convenient to the participants. *Transferability* was ensured by providing a detailed description of the study settings, research context and participants, allowing readers to determine the applicability of the findings to a similar population and context.

### Ethical considerations

Ethical approval was obtained from the Research Ethics Committee of the Faculty of Health Sciences, University of Pretoria (Ref: 341/2024), and the Ghana Health Service Ethics Review Committee (Ref: GHS-ERC:008/10/24). Administrative permissions were granted by the management of TTH, NRH and WH.

Informed consent: Participants were assured of confidentiality, voluntary participation and the right to withdraw at any stage without repercussions for their care. All identifying information was removed from transcripts, and pseudonyms were used during reporting. Audio files and transcripts were stored in password-protected devices accessible only to the research team.

## Results

### Demographics of the participants

Table 1 presents the demographic characteristics of respondents, including age, study sites, marital status, educational level, duration of diagnosis and ART status. The total number of participants who took part in this study were 20 WRA living with HIV/AIDS, with their ages ranging from 19 to 40 years, with a median age of 30 years. Participants were purposively selected from three health facilities in Northern Ghana, including TTH (40%), Tamale WH (25%) and NRH (35%). Participants represented a range of marital statuses, including single (35%), married (25%), divorced (20%) and widowed (20%). Participants’ educational status ranged from no formal education (50%) to formal education level (50%). Most of the participants had been diagnosed with HIV/AIDS for more than 1 year, and were actively attending ART clinics and taking ART medication. Interviews with WRA attending HIV clinics across the three facilities yielded a coherent account of participant-reported self-management needs, preferences and culturally embedded practices influencing self-management. Participants varied by age, marital status and time since diagnosis.

**TABLE 1:** Demographic characteristics of participants (*N* = 20).

Variable	Category	Frequency (*n*)	%
Age (years)	Range	19–40	-
	Median	30	-
Study site	Tamale Teaching Hospital	8	40.0
	Tamale West Hospital	5	25.0
	Northern Regional Hospital	7	35.0
Marital status	Single	7	35.0
	Married	5	25.0
	Divorced	4	20.0
	Widowed	4	20.0
Educational level	Formal education	10	50.0
	Non-formal education	10	50.0
Duration of diagnosis (year)	> 1	Majority	-
	≤ 1	Minority	-
ART status	On ART	20	100

ART, antiretroviral therapy.

Organising the analysis within the PEN-3 model (Relationships and Expectations), three themes captured women’s perspectives: (1) perceptions regarding needs for self-management of HIV, (2) enablers of self-management and (3) nurturers of self-management. Quotations were anonymised by site and participant number.

### Summary of the main themes of the study, subthemes and illustrative participants’ quotations, aligning relationship and expectation domains of PEN-3 cultural model

Table 2 presents a summary of the main themes of the study, subthemes and illustrative participants’ quotations, aligning relationship and expectation domains of PEN-3 cultural model. It summarises how women’s perception, enablers and nurturers influence HIV/AIDS self-management practices in Northern Ghana. Each subtheme is categorised as an enabler or barrier, which is supported with verbatim evidence from the participants, providing a brief summary of how cultural, social and psychological factors influence women’s experiences and preferences in managing their conditions.

**TABLE 2:** PEN-3 cultural (perceptions, enablers and nurturers in concerning relationships and expectation domain) alignment of themes, subthemes and illustrative evidence.

Themes	Subthemes	Description of subthemes	Direction	Illustrative evidence (verbatim)	Study site	Participant
1. Perceptions regarding needs for self-management of HIV/AIDS	1.1. Healthcare needs and service logistics	Knowledge of the HIV disease process, uninterrupted ART, nutrition education and predictable clinic processes	Enabler	‘If you don’t understand the illness you can’t manage it well … “The drugs are what keep us alive” … nutrition, access to ART drugs, no delays at the facility’.	TTH TTH	P4 P5
		Transport cost and queuing constraints	Barrier	‘I do not have money for transport … so I may miss doses’.	WH	P2
	1.2. Psychological and emotional needs	Fear of disclosure and stigma	Barrier	‘I have to hide when going to the hospital. We need psychological counselling support’.	WH	P2
		Mood and anxiety linked to scarcity	Barrier	‘Anxiety and depression come when I do not have food or money’.	TTH	P5
	1.3 Treatment and information preferences	Interest in long-acting injectables	Enabler	‘If there is an injectables, it could help us …’.	TTH	P3
		Practical education and confidential counselling	Enabler	‘I prefer education from nurses or via phone calls’.	WH	P3
2. Enablers of self-management of HIV/AIDS	2.1. Faith and cultural platforms for education and stigma reduction	Sermons and homilies with factual messages	Enabler with safeguards	‘Every Friday in the mosque, they should educate people on HIV self-care’.	TTH	P1
		Community durbars and festivals	Enabler	‘Durbars can be a source of HIV education’.	TTH	P3
	2.2. Mass media and moderated digital messaging	Local language radio and television	Enabler	‘More feasible in a TV programme’.	NRH	P2
		Misinformation and discriminatory attitudes	Risk or caveat	‘People give false testimonies that pastors have cured them’.	TTH	P2
3. Nurturers of self-management of HIV/AIDS	3.1. Family and partner support	Reminders and emotional support	Enabler	‘My husband reminded me … now I take it on my own’.	TTH	P1
	3.2. Peer mentorship and groups	Models of hope and structured groups	Enabler	‘My auntie is a model of hope person …’.	WH	P2
		Confidentiality concerns about formal groups	Barrier	‘We used to have groups … you keep thinking quietly’.	NRH	P2
	3.3. Digital nurturers and confidentiality preferences	WhatsApp reminders and encouragement	Enabler	‘We need to have a WhatsApp group, when it is 8:00 we remind each other’.	TTH	P4
	3.4. Programme sustainability	Donor fatigue limiting group activities	Barrier	‘We cannot even call a meeting. We need donor support’.	NRH	P3

TTH, Tamale Teaching Hospital; WH, Tamale West Hospital; NRH, Northern Regional Hospital.

#### Theme 1: Perceptions regarding needs and preferences for self-management of HIV/AIDS

The first theme underscores how WRA living with HIV/AIDS perceived their needs and preferences in self-managing their conditions. Their narratives reflected a strong desire for a care model that is responsive to the needs, preferences and culturally appropriate. Three subthemes were identified: healthcare needs and service logistics, psychological and emotional needs, and treatment and HIV information preferences.

**Subtheme 1.1: Healthcare needs and service logistics:** Women described and prioritised their healthcare needs of self-management in terms of both gaining access to services of ART and the quality of service delivery, which the participants viewed as distinct but interrelated. The participants prioritised uninterrupted access to ART supply as their most critical need to practise self-management. They linked transport costs, lost income and time spent in queues as contributing to missed appointments and delayed refills or missed doses, hindering their self-care abilities. Respectful interactions and coordinated services at a single visit were described as facilitators of self-management of their conditions:

‘Medically, what I just need is the antiretroviral drugs, the ART … it is only the ART that I need most.’ (NRH Participant 2, 26 years, Female)

‘I need quality service, timely access to ART drugs, no delays at the facility.’ (TTH Participant 1, 32 years, Female)

‘I need financial support. Sometimes I do not have money for transport to the hospital to get my medicine, so I may miss doses.’ (WH Participant 2, 30 years, Female)

**Subtheme 1.2: Psychological and emotional needs:** Women’s psychological and emotional needs were around the need for confidentiality, support for stigma-free and coping resources to enhance consistent self-management. Fear of disclosure and anticipated stigma shaped how participants navigate self-care, often influencing daily routines such as timing clinic visits to avoid recognition and limiting disclosure to trusted persons, all of which affected adherence to self-care practices. For instance, one of the participants said:

‘I have to hide when going to the hospital. People may ask where I am going, and I cannot tell them the truth. We need psychological counselling support.’ (WH Participant 2, 30 years, Female)

‘Sometimes the way they point fingers … the stigma affects my emotional wellbeing. I need to know stigma management strategies.’ (WH Participant 1, 35 years, Female)

‘We cannot even call a meeting. We need donor support.’ (NRH Participant 3, 29 years, Female)

Periods of anxiety and low mood were often linked to food or financial insecurity. Prayer, private counselling and reassurance from trusted others were used to cope. Participants noted that:

‘Yes, anxiety and depression come when I do not have food to eat before or after taking the medication, or I do not have money for transport to the clinic or buy food.’ (TTH Participant 5, 34 years, Female)

‘I pray to God and ask God to protect me from stigma and discrimination.’ (WH Participant 4, 24 years, Female)

**Subtheme 1.3: Treatment and information preferences:** Women identified a clear need for information that is practical and contextually relevant to support HIV self-management. Central to this were the emphasis on understanding the HIV disease process and the importance of strict adherence to ART. Participants also expressed the growing need for alternative HIV/AIDS treatment modalities, such as long-acting injectable therapies, which participants perceived as a potential solution to problems such as forgetfulness, pill fatigue and the social burden of daily use of ART. Most participants reported requesting counselling and practical education on the side effects of medication, nutrition and how to integrate treatment within daily life. One participant said:

‘If you understand the HIV diseases, you can manage it well … the medications are what keep us alive.’ (TTH Participant 4, 25 years, Female)

Another participant said:

‘Currently, I prefer that if we could get injectables and information on it, it would have been better than the oral HIV drugs, and telling us the kind of food to eat that can help me maintain good health.’ (TTH Participant 3, 38 years, Female)

One participant expressed:

‘The nurse told me that I should always make sure I adhere to the ART … I have never used herbal concoction.’ (TTH Participant 6, 31 years, Female)

‘Through radio stations and sometimes from television and through group discussion, I prefer receiving information on my condition through counselling, side effects of ART medication …’ (WH Participant 1, 35 years, Female)

‘I prefer receiving health education from the nurses or via phone calls, that is confidential.’ (WH Participant 3, 33 years, Female)

#### Theme 2: Enablers of self-management of HIV/AIDS

This theme demonstrates the structures, systems and platforms that enable WRA to effectively manage their conditions. Participants described both modern and traditional mechanisms as being supportive in maintaining adherence to treatment and resilience. Faith and cultural platforms for education and stigma reduction, mass media and moderated digital messaging emerged as powerful enablers and were viewed as trusted channels for receiving health education and counselling.

**Subtheme 2.1: Faith and cultural platforms for education and stigma reduction:** Participants identified mosques and churches as trusted venues for factual, brief messages that normalise HIV, discourage discrimination, and signpost to services. Community gatherings such as durbars (community meeting focal point) and festivals were also seen as credible opportunities for outreach. Women expressed a critical need for messages consistent with clinical guidance and privacy:

‘Every Friday in the mosque, the Imams [*leader*] should include health education in the sermons such as avoidance of stigma on people suffering from chronic diseases such as HIV/AIDS.’ (TTH Participant 1, 32 years, Female)

‘Christians, during Sunday church services, can have pastors preach against HIV stigmatisation. They should be informed that HIV does not kill and there are drugs available.’ (TTH Participant 3, 38 years, Female)

‘Cultural practices like community durbars, naming ceremonies, and festivals like Damba can be used as good platforms to educate people on HIV prevention and stigma free messages can help boost self-management practices.’ (WH Participant 1, 35 years, Female)

‘Culturally, durbars can be a source of HIV education. Nurses can be brought to the chief’s palace for testing community members.’ (TTH Participant 3, 38 years, Female)

At the same time, some participants described religious settings where stigma or misinformation could undermine adherence as an influential factor in self-management. If religion does not support people with chronic diseases, it will serve as one of the barriers to self-management practices among women:

‘In my religion, sometimes they are even the worst. If they know you are in such condition, nobody will even stand beside you again in the mosque.’ (NRH Participant 4, 38 years, Female)

‘Most of the time in churches, people give false testimonies that pastors have cured them. Meanwhile, the treatment for HIV is only ART.’ (TTH Participant 2, 19 years, Female)

**Subtheme 2.2: Mass media and moderated digital messaging:** Local language radio and television were viewed as enablers, feasible, high-reach channels to reinforce clinic education. Moderated social media, including WhatsApp groups and short clinic screen messages, were described as acceptable complements:

‘This one is more feasible in a TV programme.’ (NRH Participant 2, 26 years, Female)

#### Theme 3: Nurturers of self-management of HIV/AIDS

This theme focuses on nurturing support systems – digital and interpersonal – that sustain WRA’s efforts in self-managing their conditions. The systems were described as important means for treatment adherence, emotional well-being and enhancing self-management. Among the subthemes that emerged are: need for family and partner support, which was seen as a stabilising influence. The need for peer mentorship and groups has emerged as a significant nurturer. Digital nurturers and confidentiality preferences reflected the increasing role of technology in fostering supportive networks and HIV education.

**Subtheme 3.1: Family and partner support:** Spouses and trusted relatives helped with reminders and emotional support. Where present, supportive relationships reduced distress and were described as strengthening adherence:

‘At the beginning, my husband reminded me. Now I take it on my own at bedtime.’ (TTH Participant 1, 32 years, Female)

**Subtheme 3.2: Peer mentorship and groups:** Participants expressed a critical need for support from peer mentorship. Peer models, including models of hope, provided experiential guidance on disclosure, clinic navigation and symptom management. Structured groups were valued for sharing ideas and encouragement, although some participants preferred one-to-one contact to protect confidentiality. A participant expressed:

‘My auntie is a model of hope, and she was the one who first explained my diagnosis to me. I like receiving health education from them.’ (WH Participant 2, 30 years, Female)

Another one said:

‘Yes, we used to have groups. We share ideas and experiences. But when you are home alone, you keep thinking quietly.’ (NRH Participant 2, 26 years, Female)

**Subtheme 3.3: Digital nurturers and confidentiality preferences:** WhatsApp groups supported medication reminders at agreed times and provided encouragement on days when motivation was low or travel was difficult. Some women avoided formal groups because of confidentiality concerns or perceived reductions in donor funding, opting instead for one-to-one or digital supports that felt safer:

‘We need to have a WhatsApp group. When it is 8:00 we remind each other, it is time to take our medication.’ (TTH Participant 4, 25 years, Female)

‘Because of donor fatigue, we do not receive any support. We cannot even call a meeting; we need donor support.’ (NRH Participant 3, 29 years, Female)

**Subtheme 3.4: Programme sustainability:** Emerged as a concern affecting nurturing support. Several participants noted that donor fatigue had reduced the frequency of peer group meetings and limited ongoing group-based support: ‘Because of donor fatigue, we do not receive any support. We cannot even call a meeting; we need donor support’ (NRH Participant 3). This suggests that the sustainability of externally funded peer-support structures is itself a determinant of women’s access to nurturing support.

### Cross-cutting observations

Across themes, women stressed the value of respectful, confidential clinical encounters. Where trust was established, participants reported greater comfort asking questions, disclosing side effects and seeking help with practical obstacles. Economic vulnerability intersected with all domains. Transport subsidies, small livelihood grants or food support were frequently cited as enabling consistent self-management. Finally, the data show a pragmatic integration of supports: need for biomedical treatment anchored by the clinic, need for everyday encouragement from family and peers, and culturally salient spaces that normalise accurate information. Women articulated the need for self-management as a practical and social process that depends on reliable therapy access, stigma-aware psychosocial support strategies, actionable education and culturally credible communication channels for HIV education. Digital peer messaging via WhatsApp has become part of the adherence ecosystem, operating alongside family reminders and clinic counselling. Overall, WRA identified their needs and preferences for HIV self-management. These findings identify modifiable points for HIV programme design, including transport and scheduling supports, options for long-acting regimens, protected small-group or digital peer forums, and structured engagement of religious and community leaders in routine health communication.

## Discussion

This study explored the needs and preferences of WRA in Northern Ghana to practise HIV/AIDS self-management, and how context shapes their choices. Interpreting the findings through the *PEN-3* cultural model, specifically the *Relationships and Expectations domain*, clarified three interlocking themes: perceptions regarding needs for self-management, enablers and nurturers of self-management. This framing highlights how service logistics, information needs, social context and cultural settings interact to influence daily adherence and health-seeking behaviours (Airhihenbuwa et al. 2014).

The findings of this study highlight that culture is not an outlying influence, but a main determinant of women’s self-management capabilities, shaping how WRA interpret HIV/AIDS, engage with care and navigate stigma, while also influencing whether women feel comfortable using available support systems such as family network, community structures and religious spaces, which can either support or constrain women’s ability to adhere to HIV self-management practices. World Health Organization (2022) highlights that culture influences HIV/AIDS self-management by shaping individual beliefs, health-seeking behaviours, stigma experiences and adherence practices within their social and religious context. This highlights the importance of culturally tailored health education programmes. This study found that women expressed a critical need for uninterrupted access to ART and predictable clinic processes as the bedrock of self-management. Participants consistently highlight the need to have continuous availability of ART services and education, often describing it as the primary requirement for maintaining health and preventing deterioration of their conditions. The emphasis on having uninterrupted ART was not only limited to the medication (ART) availability within the ART clinics, but also extended to their ability to reliably access the medication without interruption. Practical barriers, including transport costs, time spent in queues and lost income on clinic days, were directly linked to delayed refills and missed doses in our data. These patterns are consistent with evidence from sub-Saharan Africa that material constraints undermine retention and adherence (Kwena et al. 2023; UNAIDS 2022; WHO 2022). Psychological and emotional needs were closely related with service access. Fear of disclosure, anticipated stigma and episodes of low mood or anxiety, often intensified by food insecurity, shaped women’s willingness to seek care and their daily medication routines. Similar dynamics have been documented in Kenya and South Africa, where stigma, financial and food scarcity constrain disclosure and continuity of care (Copeland 2018b; Gumede et al. 2022). Interest in long-acting injectable regimens reflects a desire to limit pill fatigue and the visibility of medicines, which may ease both daily burden and stigma, as reported in other settings that emphasise simplified regimens and person-centred care (O’brien et al. 2020). A study conducted in Mombasa further adds that most women living with HIV reported avoiding herbal remedies after counselling, echoing evidence that clear guidance on interactions between antiretroviral medication and herbal remedies supports safer self-management (Cheptoek et al. 2024). They also asked for practical, confidential education on side effects, nutrition and daily routines, which is in agreement with global guidance on self-care and patient literacy (WHO 2022). Even though the participants described the preferences that align closely with the global trends towards person-centred HIV treatment, a notable contrast arises as compared to the findings from studies. For instance, while many participants in this study expressed interest in long-acting injectable treatment regimens to reduce pill fatigue, lessen stigma and increase privacy, some women in other studies reported different preferences, with participants preferring oral therapy because of familiarity with oral treatment regimen (Philbin et al. 2021). In this study, the WRA living with HIV identified religious venues and community events as credible platforms for education and stigma reduction. Friday prayers, Sunday services, durbars and festivals were viewed as opportunities for delivering short, factual messages that normalise HIV, discourage discrimination, and refer women to appropriate HIV/AIDS care and support services. Prior work in the region shows that culturally resonant channels and faith leaders can extend the reach and acceptability of HIV messaging when content is accurate and respectful (Bergman et al. 2023; UNAIDS 2022). This finding is consistent with a study conducted in Kenya, which found that women also endorsed mass media in local languages and moderated digital channels as acceptable complements to clinic counselling, in line with the wider literature on community-based and mHealth approaches that improve reach and engagement (Rebchook et al. 2022). The same settings can, however, propagate misinformation or exclusion. Reports of ‘cure’ testimonies and judgemental attitudes in religious spaces illustrate how enablers can become barriers without careful partnership and message design. Similar concerns appear in studies from East Africa and beyond, reinforcing the need for scripts that are consistent with clinical guidance and for privacy-protecting language when working through public or faith settings (Areri 2021; Ashaba et al. 2024). Family members, partners and peers formed a layered ecology of support. Spousal reminders and encouragement from trusted relatives were frequently cited as practical aids to adherence. Trained peers and models of hope offered experiential guidance on disclosure and clinic navigation, consistent with the positive role of peer-anchored strategies in other African contexts (Poku et al. 2020; Zuniga et al. 2018). Women also described digital nurturers, particularly WhatsApp groups that provided timed reminders and encouragement on days when motivation was low or when travel was difficult. Evidence from East Africa suggests that such digital peer networks can enhance adherence when moderated and privacy-aware (Bazrafshani et al. 2022; Obeagu & Obeagu 2024). At the same time, some participants withdrew from clinic-linked groups because of confidentiality concerns or perceived reductions in donor-funded activity. This resonates with regional reports on the fragility of externally financed group models and underscores the value of flexible formats that allow one-to-one or digital participation according to preference and risk context (Bazrafshani et al. 2022; Poku et al. 2020).

### Strengths and limitations

Limitations include the single-region setting and the absence of role-linked identifiers in transcripts, which constrained subgroup analyses. As with all qualitative work, transferability depends on contextual fit, although the patterns observed are consistent with wider literature across the region. Likewise, the findings are limited to women attending ART clinics and may not reflect the experiences of women disengaged from care. Social desirability bias may also have influenced participants’ responses about ART adherence and use of traditional medicine. This study has several notable strengths. It employed a rigorous qualitative exploratory design underpinned by an established theoretical framework, the PEN-3 cultural model, which allowed culturally grounded interpretation of women’s perceptions, enablers and nurturers of self-management.

## Conclusion

In conclusion, WRA prioritised their needs and preferences for self-management in many ways. This study situating women’s accounts within the Relationships and Expectations domain of PEN-3 shows that self-management is strengthened when uninterrupted ART and streamlined clinic processes are combined with credible, culturally grounded communication and layered social support. Family, peers and moderated digital networks act as nurturers of self-management, while faith and community platforms can function as enablers when collaboration is structured to protect privacy and accuracy. These insights justify a person-centred bundle that integrates reliable access, practical counselling, peer mentorship and culturally resonant communication, and they identify where additional partnership or resourcing is needed to address persistent structural barriers. The configuration of perceptions, enablers and nurturers suggests a package of actions that is both culturally consonant and implementable in routine services. Priorities include the need for maintaining reliable ART supply and streamlined clinic flow, offering confidential education and practical counselling on side effects and nutrition, and expanding person-centred options such as long-acting injectables where feasible (UNAIDS 2022; WHO 2022). Partnerships with faith and cultural leaders can extend reach, provided scripts are vetted and privacy is protected. Moderated digital supports, including WhatsApp reminders and micro-lessons, can complement in-person counselling and peer mentoring, particularly for women who face travel costs or confidentiality risks (Bazrafshani et al. 2022; Rebchook et al. 2022). Where possible, linking women to savings groups or livelihood supports may buffer the economic pressures that undermine adherence (Kwena et al. 2023).
